# Regional γ Diversity of Diatoms in Mediterranean and Alpine Temporary Ponds

**DOI:** 10.1007/s00248-025-02670-6

**Published:** 2025-12-04

**Authors:** Davide Taurozzi, Massimiliano Scalici

**Affiliations:** 1 Department of Sciences, University of Roma Tre, Viale G. Marconi 446, Rome, 00146 Italy; 2 National Biodiversity Future Center (NBFC), Università di Palermo, Piazza Marina 61, Palermo, 90133 Italy

**Keywords:** Temporary pond, Diatom traits, Geographic variability, Diatom lifeforms, Alpha, beta, gamma diversity

## Abstract

Temporary ponds, characterized by periodic or intermittent hydroperiods, are globally widespread in all the biogeographical regions and host peculiar biotic communities. Here we investigated shifts in diatom community assemblages across two contrasting biogeographical regions in Italy, the Mediterranean and the Alpine. The study focused on 24 temporary ponds, with 12 ponds sampled at Castelporziano (CP) and 12 at Campo Imperatore (GS). Our results highlighted that γ diversity varied significantly between the two study sites, indicating a notably greater species richness in GS compared to CP. In GS, functional richness values were generally higher, whereas no significant differences were detected for functional distance and functional divergence. Species composition differed significantly between CP and GS indicating that the two sites host distinct communities, with species turnover (0.904) which contributed most to total beta diversity (0.926), while nestedness (0.021) was negligible. CP communities were characterized by pronounced functional clustering in specific sites while GS exhibited both clustering and slight overdispersion. However, although GS communities occupy slightly larger trait space, both regions shared most functional strategies, reflecting substantial redundancy in functional traits across the two environments. Overall, diatom communities in the GS were characterized by higher frequencies of small, mobile, low-profile, and mucilaginous-tube taxa, whereas CP ponds displayed relatively higher representation of larger or motile forms. Although our study is a starting point, large-scale analyses of diatom communities are crucial, as climate change may rapidly and irreversibly alter taxonomic and functional diversity, profoundly affecting the ecology of these temporary habitats and surrounding landscapes.

**Graphical Abstract:**

**Figure:**
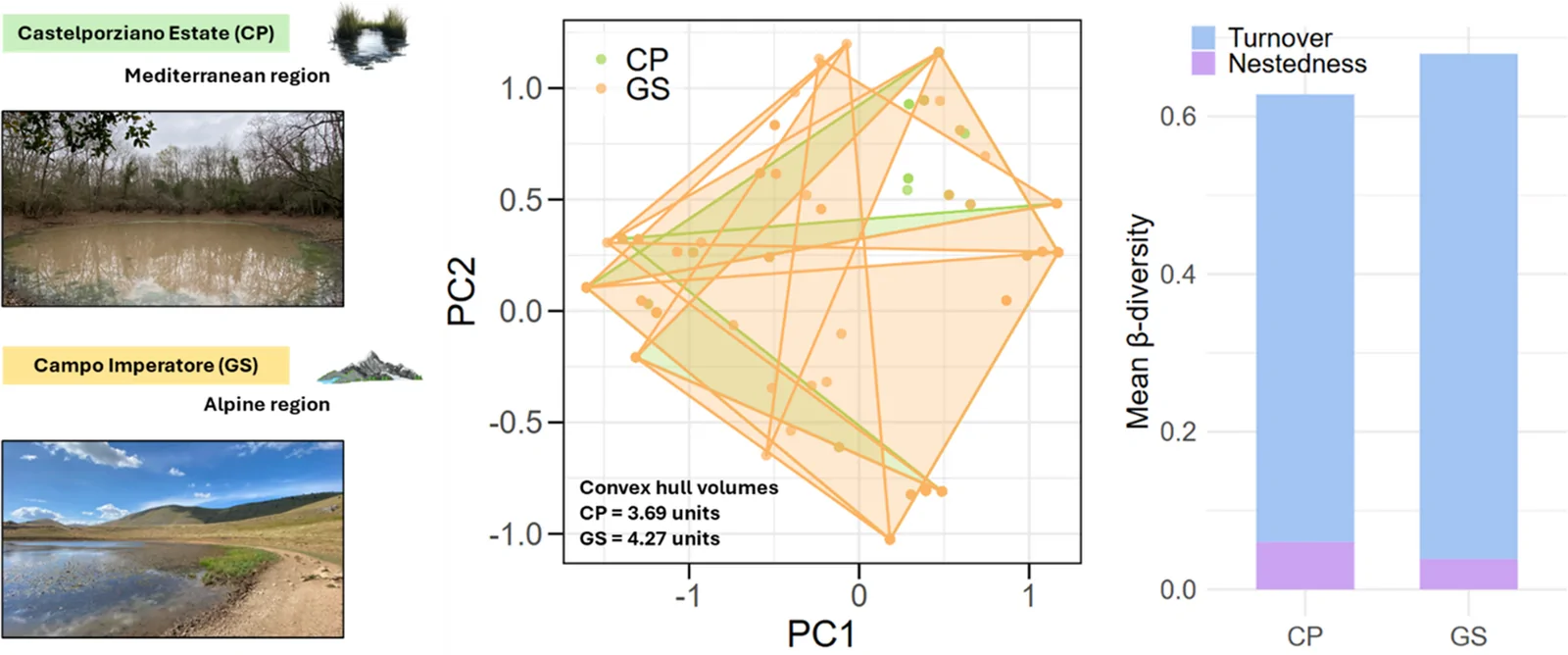

**Supplementary Information:**

The online version contains supplementary material available at 10.1007/s00248-025-02670-6.

## Introduction

Temporary ponds (hereafter TP) are unique and peculiar ecosystems, characterized by periodic hydroperiods, i.e. the alternance of wet and dry phases [1]. Although there is still no consensus on a single definition of ponds, we adopted one of the most recent definitions [2], which describes ponds as small (< 5 ha), shallow (< 5 m) waterbodies with < 30% emergent vegetation. Several factors, including light penetration, turbidity, dissolved organic matter, water supply and nutrients may influence the ecology and productivity of TP [3–5].

Hydroperiods in TP can be periodic or intermittent, depending on the presence or absence of water supply during a specific period of the year [6]. The summer season in Southern Europe, typically marked by drought and low precipitation, represents the most critical phase for TP, in particular in the Mediterranean biome [7, 8]. As a consequence, dryness excludes the more commonplace plant and animal communities, which are characteristic of more permanent waters [8]. However, not all TP show the same annual alternance of wet and dry phases. For instance, TP in Alpine biomes may experience two distinct “dry” periods: one in late summer, caused by evaporation at high temperatures, and another in winter, when ponds are either frozen or lack available water due to snow cover and low temperatures [9]. Alpine TP, also referred to as high-elevation ponds, are generally defined as shallow, small, and temporary aquatic habitats located above 300 m a.s.l [10].

Despite differences in seasonality, both Mediterranean and Alpine ponds undergo recurrent dry phases of variable duration. They are nevertheless considered key elements in landscape ecology, acting as biodiversity hotspots and reservoirs for rare species, as well as stepping stones that facilitate dispersal processes [10–13]. Furthermore, TP support entire trophic networks by providing suitable habitats for a wide range of organisms. In particular, they host primary producers, which form the basis of ecosystem structure and functioning [14–16].

Among primary producers, diatoms represent a driving force of primary production in aquatic ecosystems [17, 18]. Diatoms are unicellular algae capable of contributing approximately 20–25% of all dissolved oxygen to global net production [19]. Each species is characterized by a highly resistant silica skeleton, which enables accurate taxonomic identification [20, 21]. These characteristics, in addition to being ubiquitous, easy to sample, and each characterized by an optimum of nutrients and chemical-physical parameters, make them excellent bioindicators [22]. For these reasons, they were included in the Water Framework Directive as one of the five most recommended bioindicators for environmental monitoring [23, 24]. Despite their ecological importance, diatoms are still primarily studied in permanent aquatic systems, while their role in ephemeral waters remains largely underexplored [9]. Nevertheless, like other aquatic microorganisms, diatoms display unique adaptations to temporary habitats, including motility, specialized substrate attachment strategies, and the ability to withstand unfavorable seasons [25].

These adaptations are also shaped by larger-scale factors, including the physical, spatial, and geographical characteristics of the environmental context in which these communities occur. Comparing the same habitat across different biogeographical zones can help identify the main drivers of diatom diversity [26]. Gamma diversity (γ) represents the level of diversity observed when considering the set of communities within a broader area, linking local diversity (alpha, α) with differentiation among communities (beta, β) [27, 28]. In other words, it reflects the overall richness of species and functional traits within an ecological landscape [29]. For TP, which are often isolated and highly heterogeneous habitats [30], γ diversity provides insights into how local processes of colonization, extinction, and adaptation fit into a larger framework governed by climatic, geographical, and hydrological factors. This approach is particularly valuable when comparing Alpine and Mediterranean ecosystems. While algal communities in each pond reflect specific local conditions [31], their diversity patterns reveal the broader ecological and biogeographic variability of these regions. The Mediterranean biome is characterized by a warm, arid climate with strong hydrological seasonality [7, 32], whereas the Alpine biome features cold conditions and marked altitudinal gradients, which differentially influence the structure and composition of aquatic communities [33, 34]. In these contrasting biogeographical contexts, diatom communities are primarily shaped by a combination of abiotic and biotic drivers acting at both local and regional scales. While temperature and hydrological seasonality define the general ecological framework, other factors such as nutrient availability, conductivity, pH, light regime, and habitat stability could exert stronger and more immediate control on diatom distribution patterns [35–37]. In Mediterranean ponds, recurrent desiccation, high irradiance, and elevated ionic content often lead to community assemblages dominated by taxa tolerant to desiccation and osmotic stress [38]. Conversely, Alpine ponds are influenced by lower temperatures, reduced nutrient concentrations, and strong altitudinal gradients, which favor species adapted to oligotrophic and cold-water conditions [39]. Such differences in community structure and function are expected to become even more pronounced under future climate change, driven by rising temperatures, shifting hydrological regimes and nutrient availability and the intensification of extreme events [40–42].

From a global perspective, the conservation of TP is critically overlooked, mainly because their ecological recognition has historically been neglected. Moreover, their degradation and disappearance have steadily increased over the past centuries [11]. Analyzing shifts in diatom community assemblages across different biogeographical contexts represents a crucial first step toward understanding how future climate change may reshape these communities under contrasting environmental conditions. To our knowledge, this study represents the first attempt to investigate variations in γ diversity, defined as the total species diversity within a region or landscape, of diatom communities across TP situated within Mediterranean and Alpine biomes. In particular, we focus on how differences in environmental and biogeographical settings shape community composition and ecological functioning. Highlighting these patterns is urgent, as TP are increasingly threatened by climate change and anthropogenic pressures, and understanding their ecological dynamics is essential to anticipate future biodiversity responses and to guide effective conservation strategies.

## Materials and Methods

### Study Area and Sampling Activities

**Fig. 1:**
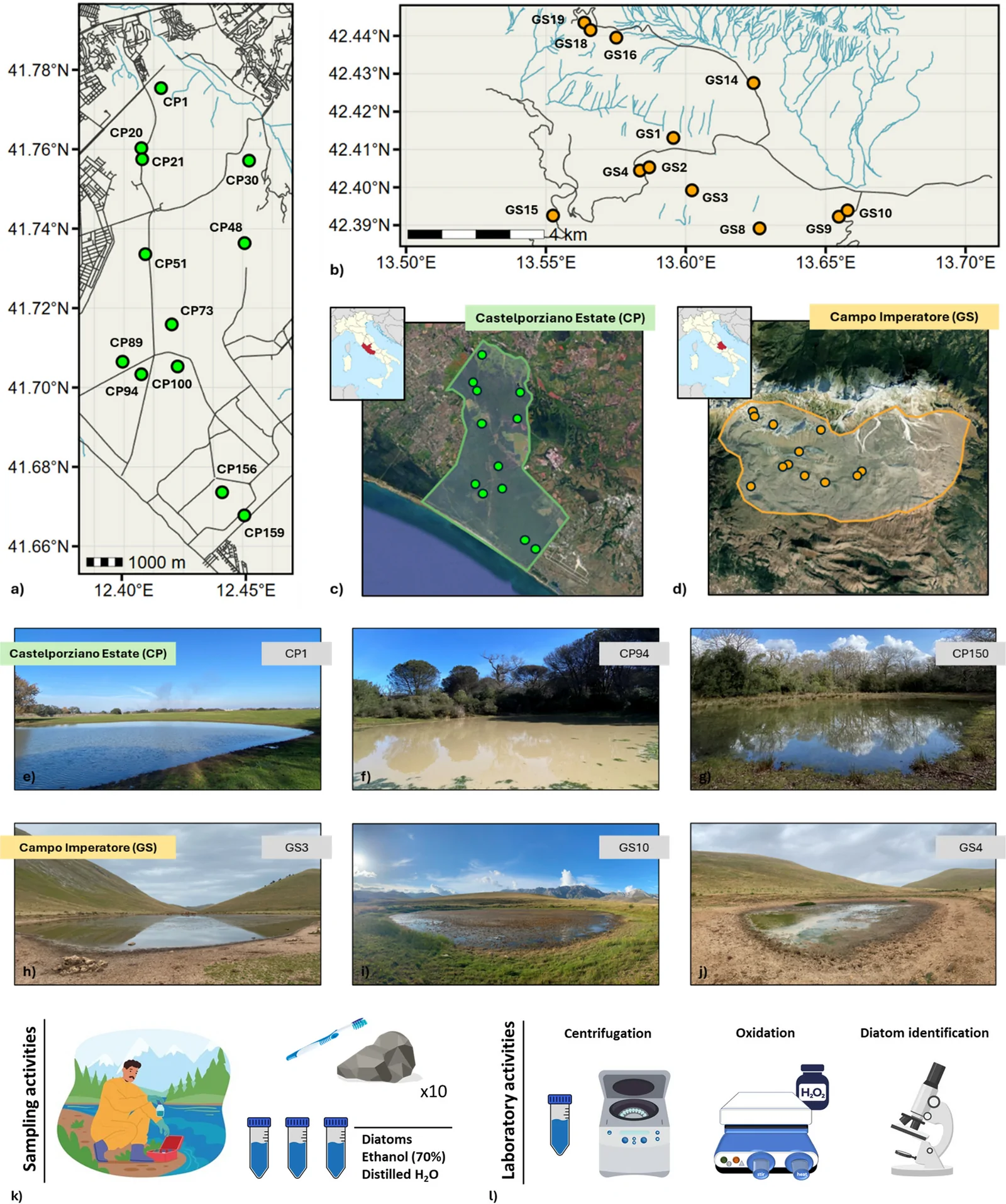
** a**) Map of sampled ponds in the Castelporziano estate and **b**) in the Campo Imperatore plateau; **c**) geographical distribution of TP in the Castelporziano estate and d) in the Campo Imperatore plateau. Field photos taken by the authors of sampled ponds: **e** - **g**) Castelporziano estate; **h** – **j**) Campo Imperatore plateau. **k**) schematic representation of sampling activities and **l**) laboratory activities for diatom oxidation and identification

The two study areas considered are the Presidential Estate of Castelporziano, representative of the Mediterranean biogeographic region, and the Campo Imperatore plateau, within the Gran Sasso and Monti della Laga National Park, representative of the Alpine region. At each site, 12 temporary ponds (TP) were sampled, for a total of 24 ponds (Table S1 (Fig. 1a, b, c and d)). Pond temporary hydroperiod was derived from both in situ observations and satellite analyses [43, 44].

The Castelporziano Presidential Estate (hereafter CP, 41°44’37"N 12°24’00"E) lies approximately 25 km from the center of Rome and covers an area of 60 km^2^ (6,039 hectares), with altitude ranging from 0 to 78 m a.s.l. The estate is largely dominated by hygrophilous lowland forest, composed mainly of evergreen and deciduous oaks, together with more strictly hygrophilous species near wetlands. Other typical environments include coastal dunes, pine forests, cultivated fields and areas used for free-range livestock farming. Ponds of the estate represent remnants of ancient humid environments, flooded forests, and marshes that once extended across a much larger and continuous landscape (Fig. 1e and f, 1 g). Access to the estate is restricted to authorized staff and researchers.

The Campo Imperatore plateau (hereafter GS, 42°24’41"N 13°37’30"E) extends for approximately 20 km in length and 3–7 km in width, with elevations ranging from 1,500 to 2,000 m a.s.l., and is surrounded by the highest peaks of the Apennines, including Corno Grande (2,912 m) and Monte Camicia (2,564 m). The plateau is of glacial and karst-alluvial origin and is almost completely devoid of vegetation, due both to its high altitude and to centuries of deforestation and is currently characterized by extensive wild grazing. Annual precipitation averages around 900 mm, with peaks in autumn. Snow cover persists for most of the year, generally from November to May, and its melting supplies water to natural basins that give rise to TP (Fig. 1 h, i and j).

The wet hydroperiod of Castelporziano ponds extends from October to May, with peak precipitation in January and February; during the boreal summer, scarce precipitation leads to a prolonged dry phase. In contrast, Campo Imperatore TP exhibit two distinct “dry” periods: one during the winter period, from October to April, when ponds are covered by snow and ice, and another during August and early September, when high temperatures and limited precipitation cause complete desiccation. To ensure sampling during the most suitable periods, diatoms were collected in CP in February, corresponding to the maximum of the wet hydroperiod and in GS in July 2024, representing the late stage of the hydroperiod, when ponds are still water-filled but approaching desiccation. The choice of sampling in GS during summer was also constrained by logistical challenges (snow, closed roads) typical of high-altitude sites, which prevent access outside the summer season. This timing was chosen to allow diatom communities to reach a certain degree of stability while avoiding late spring–early summer, when high concentrations of suspended solids can substantially alter community structure [45].

Diatoms were sampled following standard methodologies [45]: For each sample, at least 10 stones (dimensions between 64 mm and 256 mm) were collected exclusively from the shoreline, at a maximum distance of 1 m from the water’s edge and scraped using a commercial toothbrush; then, the toothbrush was immersed in a 50 ml Falcon containing 70% diluted ethanol and distilled water (Fig. 1k). Once in the laboratory, diatom samples were oxidized following the hydrogen peroxide method [45]. Briefly, 5 ml of sample were centrifuged at 1500 rpm for 10 min, the supernatant was discarded, and the sediment was mixed with 20 ml of H_2_O_2_ and heated for 3 h to degrade organic matter (Fig. 1 l). After complete oxidation, the mixture was centrifuged three times for 10 min each, and diatoms were mounted on permanent slides using Naphrax^®^ (high refractive index resin) to fix the coverslip. For each site, three different replicates were performed [46]. Diatom identification was performed using a Leica microscope at 100× magnification, with the help of specific taxonomic guides [47–54]. For each slide, the analysis was considered completed when identification at the species level of 400 valves was achieved.

### Data Analysis

#### Taxonomic and Functional α and γ Diversity

Taxonomic α diversity was calculated for each site and across all sites combined using the *specnumber()* function from the *vegan* R package [55]. Site-level species richness was then compared between Castelporziano and Campo Imperatore using a Wilcoxon rank-sum test (wilcox.test() in base R). To complement the statistical significance, we calculated the effect size using Cliff’s delta (cliff.delta() function from the *effsize* R package) [56]. Cliff’s delta is a non-parametric measure of effect size that estimates the probability that a randomly chosen value from one group is larger than a randomly chosen value from another group, minus the reverse probability [57]. In contrast, γ diversity was computed as the total number of species pooled across all ponds within each study area.

To evaluate species accumulation and assess differences in taxonomic richness between sites, species accumulation curves were computed for each study area using the *specaccum()* function from the *vegan* R package [55]. These curves describe the cumulative number of species observed as sampling effort (i.e., number of ponds) increases. To quantify overall species richness across all sampling units, we calculated the area under the accumulation curve (AUC) by summing the cumulative richness values for each site. Differences in cumulative richness between Castelporziano and Campo Imperatore were tested using a permutation approach: pond labels were randomly reassigned across both study areas 10,000 times, species accumulation curves were recalculated for each permutation, and the difference in AUC between permuted groups was recorded. This allowed us to test whether the observed difference in cumulative richness could arise by chance when ponds are freely shuffled between sites. The observed difference in AUC was then compared against the null distribution to obtain a one-tailed p-value, providing a statistical test of whether observed species accumulation differed significantly from random expectations.

Diatoms were classified according to three functional trait categories: size classes, ecological guilds, and life-forms [58–60] (Table S2). Size classes refer to the categorization of diatoms based on their cell size, which influences ecological processes such as growth rate and sedimentation. Ecological guilds group diatoms based on their adaptive strategies to environmental factors like nutrient availability and disturbance, reflecting their ecological roles within the community. Life-forms describe the morphological and behavioral traits related to the diatoms’ mode of attachment or mobility, indicating their adaptations to specific microhabitats.

Functional α diversity was calculated for each site and across all sites combined using the *dbFD()* function from the *FD* R package [61]and. Prior to calculation, trait matrices were filtered to include only species present at each site. Principal Component Analysis (PCA) was applied to the trait matrices (*prcomp()* in base R) to reduce dimensionality, retaining up to 10 axes per site depending on species richness. The PCA scores were not interpreted directly but served as input for the subsequent computation of functional indices, ensuring that correlated traits did not bias the metrics. Site-level functional richness (FRic), functional dispersion (FDis), and functional divergence (FDiv) were then calculated for both sites. Functional richness (FRic) quantifies the volume of the functional trait space occupied by a community, reflecting the range of traits present. Functional evenness (FDis) describes the regularity in the distribution of species abundances and their functional dissimilarities within the trait space, indicating how evenly functional traits are represented. Functional divergence (FDiv) measures the degree to which species are spread out in trait space, capturing the extent of trait differentiation among co-occurring species. Total functional diversity across all sites was computed by combining site matrices. Differences between the two study areas for each functional metric were tested using Wilcoxon rank-sum tests (*wilcox.test()* in base R).

To evaluate γ diversity accumulation with increasing sampling effort, species rarefaction curves were generated for each site using the *specaccum()* function from the *vegan* package [55]. For functional rarefaction, cumulative functional diversity metrics, FRic, FDis and FDiv were calculated iteratively for increasing subsets of sampling units (ponds). At each iteration, only species present in the subset were retained, and functional diversity metrics were computed using the *dbFD()* function from the *FD* package [61]and. This procedure was repeated 100 times with randomized order of sampling units to obtain mean and standard deviation of cumulative FRic, FDis, and FDiv for each site. All functional diversity metrics were weighted by species abundance. Differences between Castelporziano and Campo Imperatore were statistically assessed by computing the area under the accumulation curve (AUC) for each metric and performing a permutation test with 10,000 randomizations, thereby testing whether the observed differences in cumulative functional diversity between sites were greater than expected by chance.

#### Compositional and Functional Analysis

Taxonomic dissimilarities among sites were calculated using the Bray-Curtis distance with the *vegdist()* function from the *vegan* package [55]. Non-metric multidimensional scaling (NMDS) was then performed with *metaMDS()* function from *vegan* package to visualize differences in community composition (k = 2). To statistically test for compositional differences between sites, we applied permutational multivariate analysis of variance (PERMANOVA) using *adonis2()* function from *vegan* package with 999 permutations. In addition, we assessed within-site dispersion (β dispersion) with *betadisper()* function from *vegan* package and tested for significant differences in variability among sites using *anova()* function. β diversity was further decomposed into turnover and nestedness components by converting the abundance matrix to presence-absence and applying functions from the *betapart* R package (*betapart.core()*, *beta.multi()*, *beta.pair()*), providing insights into the relative contributions of species replacement and loss to overall β diversity. To further explore the mechanisms underlying community assembly, we calculated Raup–Crick β-diversity (βRC) using presence-absence data. This metric provides a null-model framework to distinguish whether observed species turnover is primarily driven by deterministic (environmental filtering) or stochastic (neutral) processes. Presence-absence matrices for Castelporziano (CP) and Campo Imperatore (GS) were first aligned to include the same set of species across sites and then combined into a single matrix. Raup–Crick dissimilarities were calculated using the *raupcrick*() function from the *vegan* package with 999 randomizations. Resulting βRC values range from 0 to 1, where values close to 0.5 indicate turnover consistent with stochastic assembly, values near 1 indicate strong deterministic turnover, and values near 0 suggest lower than expected turnover. Mean and standard deviation of βRC were computed across all pairwise pond comparisons to summarize the degree of stochastic vs. deterministic influences on diatom community structure.

To explore functional community structure and assembly processes, we first calculated standardized effect sizes of mean pairwise functional distances (SES.MPD) using the *ses.mpd()* function from the *picante* R package [62], based on species abundances and functional trait dissimilarities computed with *gowdis()* from the *FD* package [61]and. Standardized effect sizes of mean pairwise functional distance (SES.MPD) were computed to quantify the functional assembly of diatom communities and to assess deviations from random expectations under a null model based on taxa labels. Negative SES.MPD values indicate functional clustering, suggesting environmental filtering or niche convergence, whereas positive values indicate functional overdispersion, potentially reflecting competitive exclusion or limiting similarity. This approach allowed us to test whether observed functional structure deviated from null expectations generated by a taxa-label randomization model (null.model = “taxa.labels”, 999 runs). Functional dissimilarities among sites were quantified by calculating pairwise Gower distances between species traits (*gowdis()* function) and computing functional diversity indices, including FRic, FDis and FDiv, with the *dbFD()* function from *FD* package. To assess the extent of functional trait space overlap between sites, principal component analysis (PCA) was performed on all species traits using *prcomp()*, retaining the first three principal components. Convex hulls enclosing species positions in the 3D PCA trait space were computed with the *convhulln()* function from the *geometry* R package [63], and their volumes were used to calculate a functional overlap index:

1 $$\\\begin{array}{c}Functional\;Overlap\;Index\;=\\\left(V_{cp}+V_{gs}-V_{combined}\right)/V_{combined}\end{array}$$

where V_CP_​ and V_GS_​ are the convex hull volumes of Castelporziano and Campo Imperatore communities, respectively, and V_combined_​ is the volume of the combined species set. An overlap index of 0 indicates no functional overlap, while 1 indicates complete overlap. To test whether the observed overlap differed from random expectation, we performed a permutation test with 999 randomizations of species identities across sites and calculated a one-tailed p-value as the proportion of permuted overlaps equal to or exceeding the observed value.

#### Indicator Species and Traits Analysis

To identify species strongly associated with each site, we applied the indicator species analysis using the *multipatt()* function from the *indicspecies* R package [64], with 999 permutations. This method calculates the association between species presence-absence patterns and site groups, highlighting species that serve as indicators of a particular site. For functional traits, species-by-trait matrices were first converted to binary presence-absence form. Site-specific mean trait values were then calculated by multiplying the binary species occurrence matrices by the trait matrix and averaging across species. This procedure provides a summary of trait prevalence per site, allowing the identification of traits that are characteristic of each community.

#### Species-Trait Network Analysis

To explore the relationships between species and their functional traits at each site, bipartite species-trait networks were constructed. In these networks, species and traits are represented as two distinct sets of nodes, and edges connect species to the traits they possess. Species-by-trait occurrence matrices were first converted to binary form and multiplied by the binary trait matrices to establish connections. Networks were then represented as graphs using the *igraph* R package [65]. Network structure was characterized by calculating node degrees (number of connections per species), modularity using the Louvain clustering algorithm (*cluster_louvain()* function), and network density (*edge_density()* function), providing measures of community connectivity and compartmentalization [66]. These metrics allowed the identification of highly connected species (“hub” species) and comparison of overall network structure between the two sites. To test whether observed modularity values were higher than expected by chance, a permutation procedure was applied. Specifically, species–trait matrices were randomized 999 times using the *r2dtable()* function (base R) to preserve row and column sums, generating null networks. Modularity was recalculated for each null network, and empirical p-values were obtained by comparing observed modularity to the distribution of null modularity.

All statistical analyses were performed using R software [67], and significance was assessed at *p* < 0.05.

## Results

### Taxonomic and Functional α and γ Diversity

**Fig. 2:**
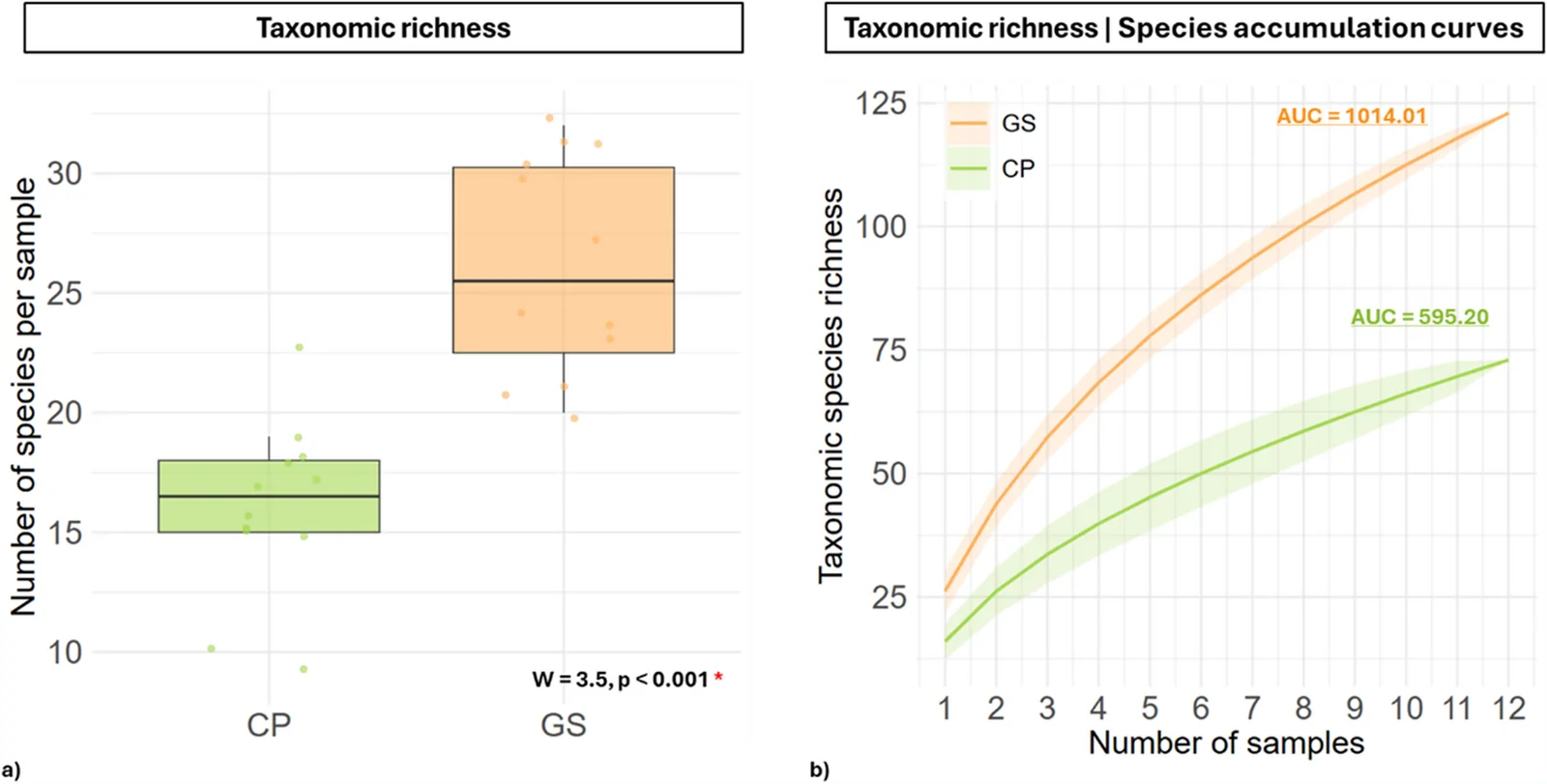
Taxonomic αdiversity across the two study sites. (**a**) Boxplot of species richness per sample at Castelporziano (CP) and Campo Imperatore (GS), showing individual sample points. (**b**) Cumulative increase (γ diversity) of taxonomic species richness across 12 sampling steps at the two sites, with mean values represented by lines and variability shown as shaded ribbons (± SD). Colors correspond to sites: CP (green) and GS (orange)

Taxonomic α diversity varied significantly between the two study sites. In Castelporziano (CP) (Table S3) the mean α diversity per pond was 16.0 ± 3.77 species, while in Campo Imperatore (GS) (Table S4) it was higher, with 26.17 ± 4.49 species per pond (Table S5 - S6). Wilcoxon rank-sum test confirmed that this difference was statistically significant (W = 3.5, *p* < 0.001), indicating a notably greater species richness in GS compared to CP (Fig. 2a). When all ponds from both sites were combined, the total α diversity encompassed the full range of observed species across the study (23–32 species per pond). The effect size, calculated using Cliff’s delta, was large and negative (δ = −0.95), confirming that samples from CP generally had lower species richness compared to GS.

Species accumulation curves (γ diversity) revealed marked differences in taxonomic richness between CP and GS. The cumulative species richness across all samples, measured as the area under the accumulation curve (AUC), was substantially higher in GS (AUC = 1014.01) than in CP (AUC = 595.20), with an observed difference of −418.81 (Fig. 2b). A permutation test with 10,000 randomizations confirmed that this difference was highly significant (*p* = 0.0002), indicating that the higher species richness in GS is unlikely to arise by chance. These results suggest that the diatom community in GS is more diverse and exhibits faster species accumulation with increasing sampling effort compared to CP.

**Fig. 3:**
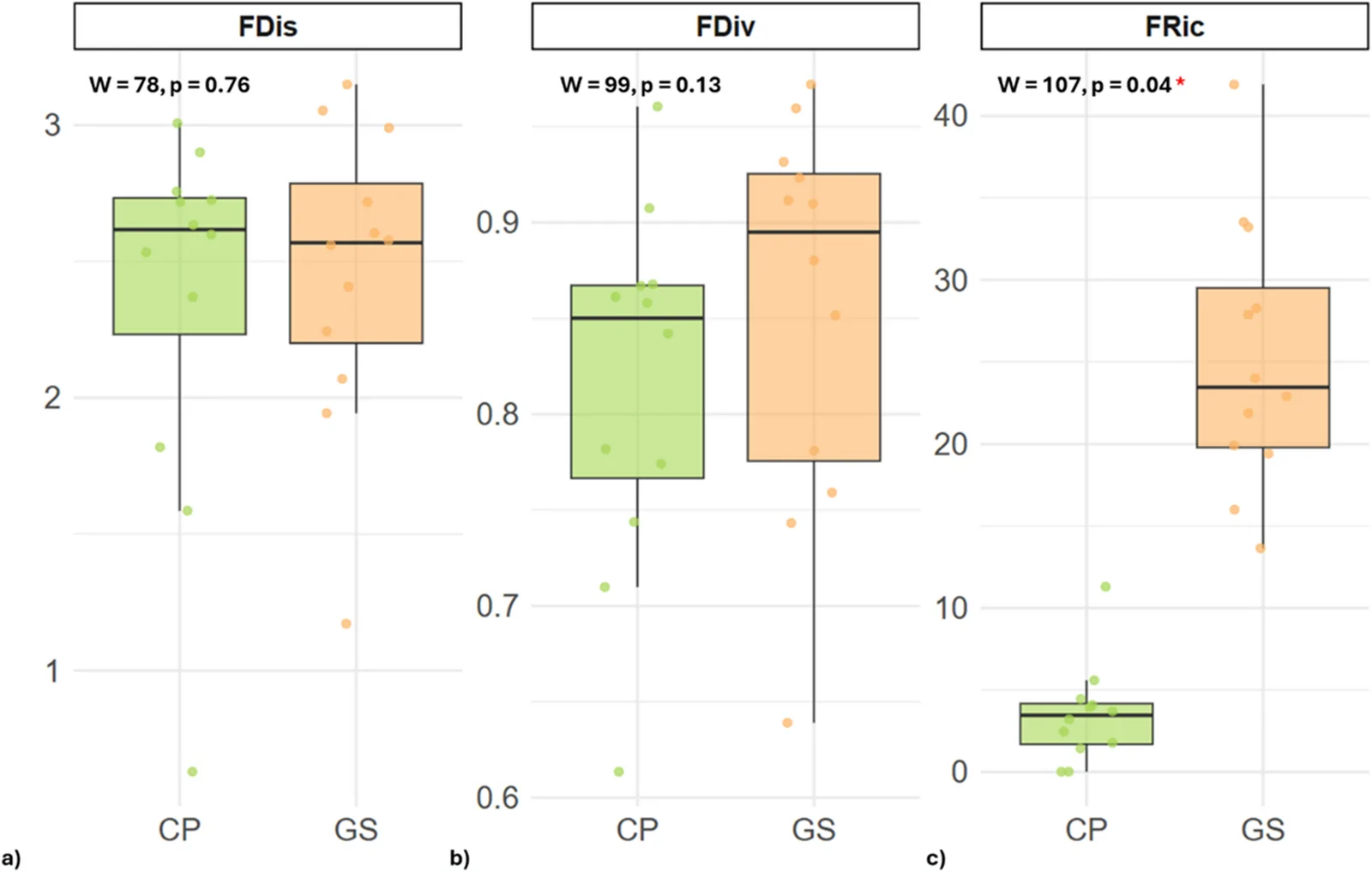
Functional diversity metrics (**a**, FDis; **b**, FDiv; **c**, FRic) across the two sites, Castelporziano (CP) and Campo Imperatore (GS). Boxplots show the distribution of values for each metric, highlighting differences in functional richness, dispersion, and divergence between sites. Colors indicate site identity: green for CP and orange for GS

Functional α diversity metrics showed differences between the two study sites (Table S6). In CP, Functional Richness (FRic) ranged from 0.01 to 14.47 across ponds, Functional Dispersion (FDis) ranged from 0.63 to 3.01, and Functional Divergence (FDiv) ranged from 0.75 to 0.97 (Fig. 3). In GS, FRic values were generally higher, ranging from 0.02 to 7.40, FDis ranged from 1.16 to 3.04, and FDiv from 0.78 to 0.96. Wilcoxon rank-sum tests indicated that FRic differed significantly between sites (W = 107, *p* = 0.04), whereas no significant differences were detected for FDis (W = 78, *p* = 0.76) or FDiv (W = 99, *p* = 0.13). These results suggest that while the number of functional traits present per pond was higher in GS, the overall distribution and divergence of traits within ponds did not differ significantly between sites.

Functional γ diversity accumulation across sites was quantified using the area under the curve (AUC) of cumulative FRic, FDis and FDiv. The results indicated a marked difference in FRic between the two study areas: CP exhibited a substantially higher cumulative FRic (AUC = 70.06) compared to GS (AUC = 21.30), with an observed difference of 48.76. Permutation test confirmed that this difference was statistically significant (*p* = 0.0059), suggesting that the species in CP occupy a wider range of functional trait space. In contrast, FDis and FDiv showed minimal differences between the two areas (AUC FDis: CP = 28.28, GS = 29.10, *p* = 0.7943; AUC FDiv: CP = 10.93, GS = 10.56, *p* = 0.2170), indicating that the overall trait dispersion and divergence were similar. These results suggest that while the breadth of FRic differs significantly between the sites, the distribution and divergence of traits within the communities are largely comparable.

### Compositional and Functional Analysis

**Fig. 4:**
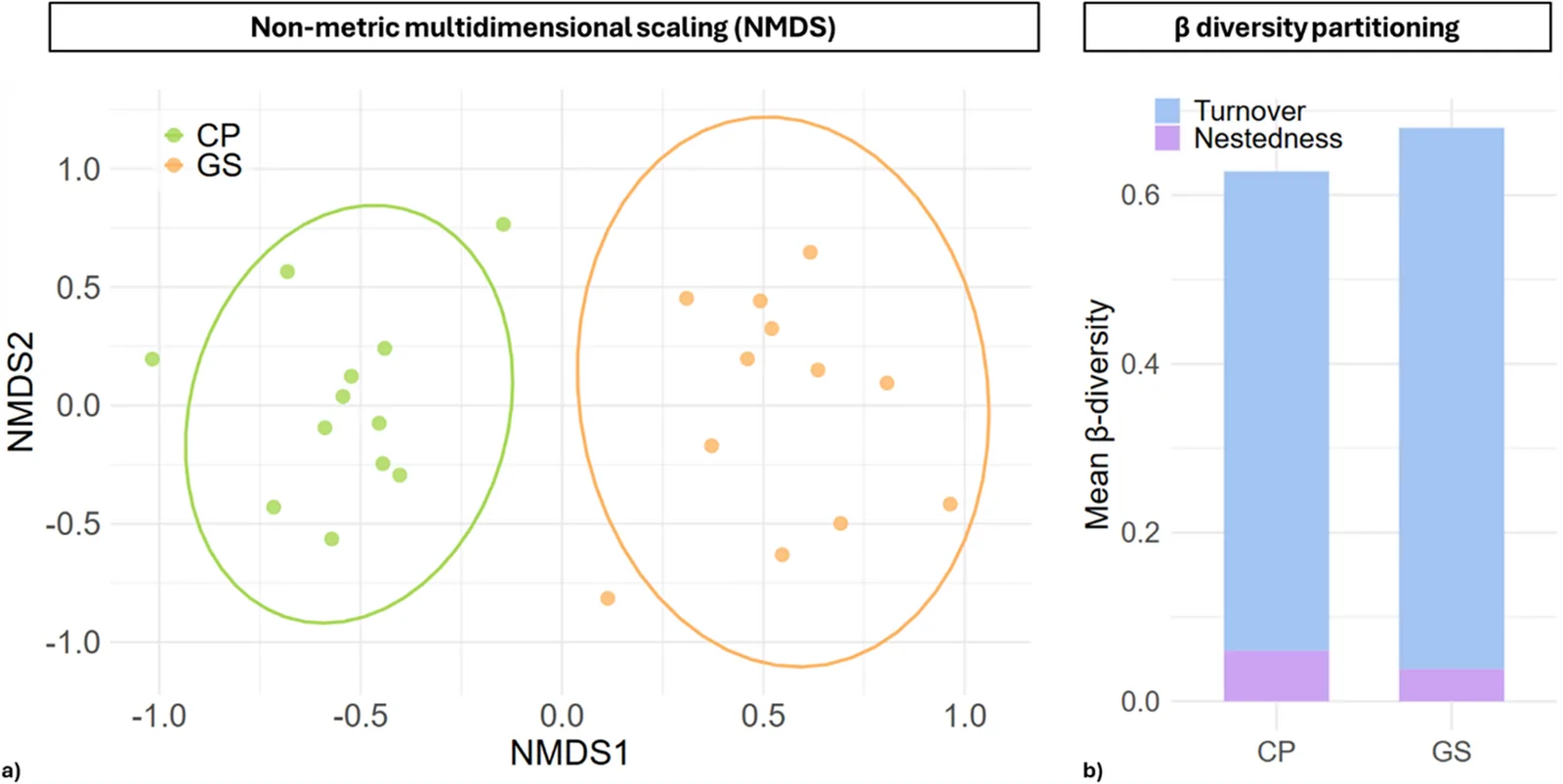
(**a**) Non-metric multidimensional scaling (NMDS) plot showing the compositional differences in diatom communities between the two sites: Castelporziano (CP, green) and Campo Imperatore (GS, orange). Each point represents a sample, and the 95% confidence ellipses highlight the multivariate dispersion within each site. (**b**) Stacked bar plot illustrating the partitioning of β diversity within each site (CP and GS) into turnover (SIM, blue) and nestedness (SNE, purple) components. The values represent the mean pairwise dissimilarities among samples within each site

Species composition differed significantly among sites within each study area (PERMANOVA, R² = 0.179, F = 4.788, *p* = 0.001), indicating that the two sites host distinct communities (Fig. 4a). Despite this, the dispersion of communities within sites did not differ significantly (betadisper, F = 0.309, *p* = 0.584), suggesting that the variability among ponds is similar within each site. β diversity decomposition revealed that species turnover (β SIM = 0.904) contributed most to total beta diversity (β SOR = 0.926), while nestedness (β SNE = 0.021) was negligible (Fig. 4b). Raup–Crick β-diversity (βRC) values, which account for stochastic assembly processes, averaged 0.56 (SD = 0.35) between ponds across the two sites. These intermediate βRC values indicate that turnover is influenced both by deterministic environmental filtering, reflecting site-specific conditions and local adaptation, and by stochastic processes such as random dispersal or ecological drift. Ecologically, this implies that differences between sites are primarily driven by replacement of species rather than loss or gain of subsets of species, reflecting site-specific environmental conditions at CP and GS, while variability within each site is partly shaped by neutral assembly mechanisms.

**Fig. 5:**
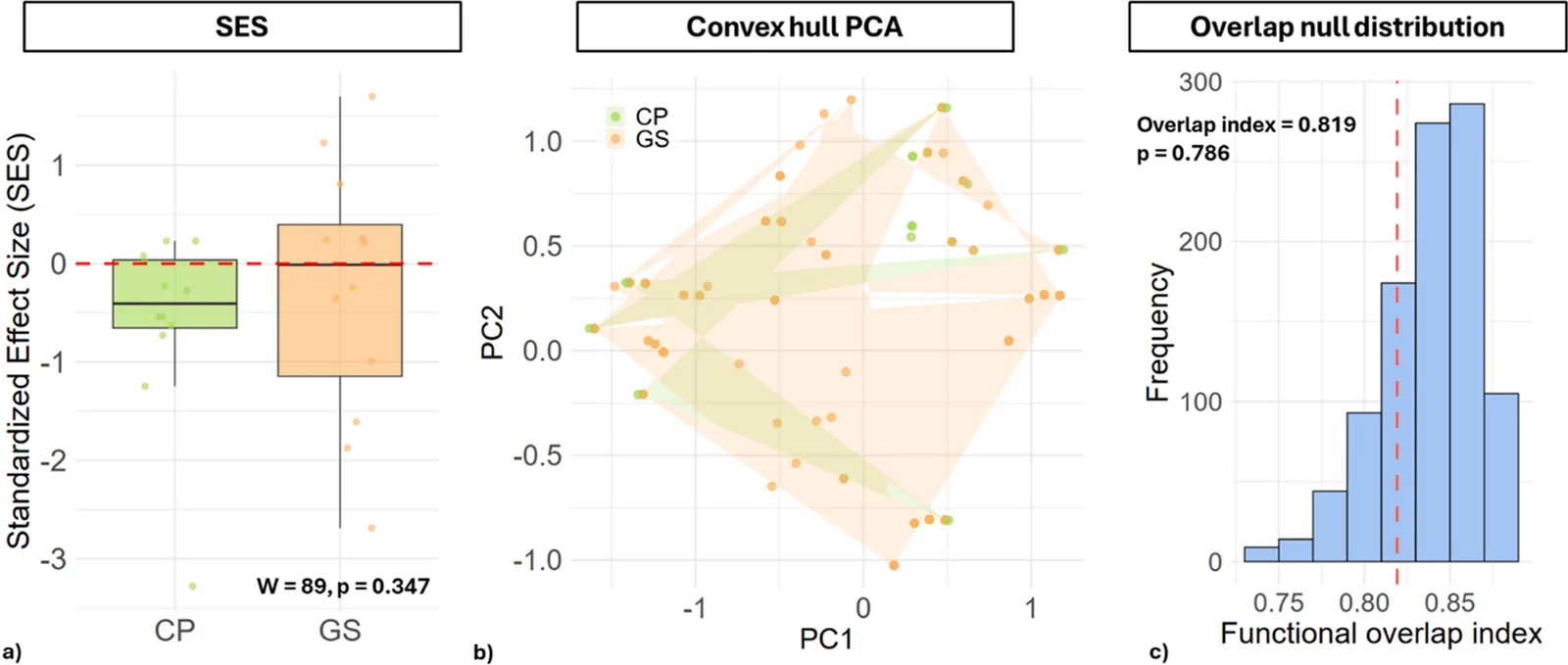
(**a**) Standardized Effect Size (SES) per site, showing the deviation of observed functional diversity from null expectations. Points represent individual samples, and the dashed red line indicates SES = 0. (**b**) Functional trait space of species in Castelporziano (CP, green) and Campo Imperatore (GS, orange) based on the first two principal components of the PCA. Each point represents a species. Transparent polygons (convex hulls) show the functional space occupied by each site. Areas where the hulls overlap indicate shared trait combinations, while non-overlapping regions highlight functional differentiation between the sites. The size and shape of each hull reflect the diversity of trait combinations within that community. (**c**) Null distribution of the functional overlap index between CP and GS derived from 999 permutations. The dashed red line represents the observed overlap, indicating the degree of functional trait sharing relative to random expectation

In CP, SES.MPD values revealed pronounced functional clustering in specific sites (e.g., SES = − 3,61), although only 1 out of 12 sites reached statistical significance (*p* < 0,05) (Table S7). In contrast, Campo Imperatore exhibited a broader range of SES.MPD values, with both clustering (minimum SES = − 2,74) and slight overdispersion (maximum SES = 1,72) observed across sites; 2 out of 12 sites showed significant functional clustering (*p* < 0,05). Despite these local patterns, a Wilcoxon rank–sum test comparing SES.MPD values between the two regions did not reveal a statistically significant difference (W = 89, *p* = 0,347), indicating that, at the regional scale, the overall functional structure was comparable between CP and GS (Fig. 5a). These results suggest that functional trait distributions are largely similar across sites, but that local environmental constraints can produce site-specific clustering, highlighting the importance of fine-scale environmental heterogeneity in shaping community functional structure.

Functional trait space was compared between CP and GS using convex hull volumes calculated on the first three principal components of the species trait matrix. CP communities spanned a convex hull volume of 3.69 units, while GS communities covered 4.27 units (Fig. 5b). The combined convex hull volume was 4.37 units, resulting in a functional overlap index of 0.819, indicating a high degree of shared trait space between the two regions (Fig. 5c). A permutation test revealed that this observed overlap was not significantly different from random expectations (*p* = 0.786), suggesting that the functional trait spaces of coastal and mountain communities largely coincide, with only moderate differentiation (Fig. 5c). Overall, these results indicate that although GS communities occupy slightly larger trait space, both regions share some functional strategies, reflecting partial redundancy in functional traits across the two environments.

### Indicator Species and Traits Analysis

**Fig. 6:**
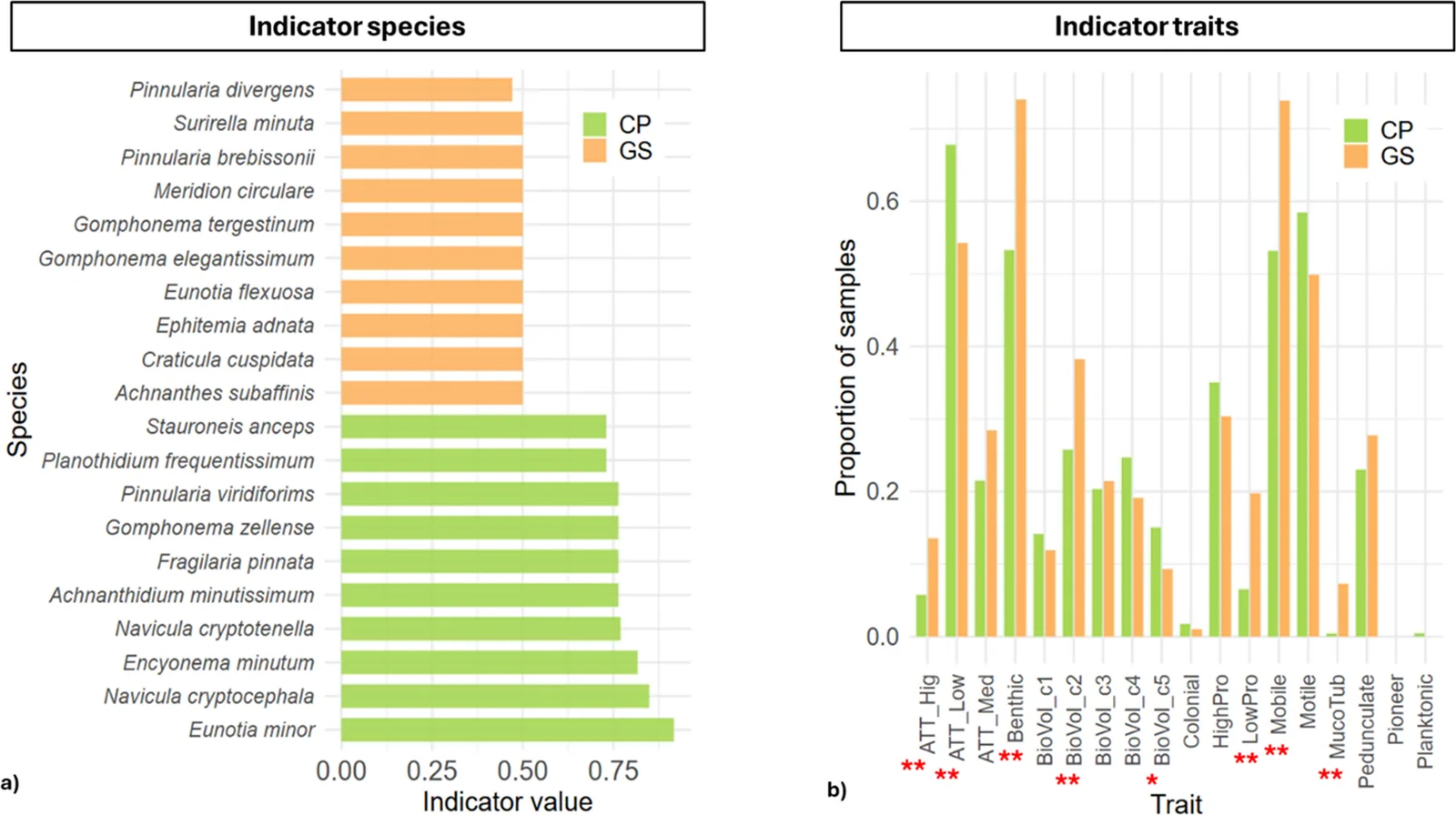
(**a**) Indicator values (IndVal) of the top species for the two study sites (CP and GS); (**b**) Mean proportion of samples exhibiting each trait across the two study sites (CP and GS). *p < 0.05, **p < 0.01

Indicator species analysis revealed 19 species significantly associated with the two sites (Table S8, Fig. 6a). GS hosted 13 indicator species, including *Achnanthidium minutissimum* (0.764, *p* = 0.007), *Cocconeis placentula* (0.645, *p* = 0.033), *Encyonema caespitosum* (0.645, *p* = 0.044), *E. mesianum* (0.707, *p* = 0.016), *E. minutum* (0.816, *p* = 0.004), *Staurosirella pinnata* (0.764, *p* = 0.005), *Gomphonema micropus* (0.707, *p* = 0.016), *Navicula cryptocephala* (IndVal = 0.849, *p* = 0.003), *N. cryptotenella* (0.770, *p* = 0.010), *N. trivialis* (0.645, *p* = 0.034). *N. veneta* (0.707, *p* = 0.014), *Planothidium frequentissimum* (0.730, *p* = 0.046) and *Fallacia subhamulata* (0.645, *p* = 0.037). CP was associated with six indicator species: *Eunotia bilunaris* (0.645, *p* = 0.038), *E. minor* (0.917, *p* < 0.001), *Gomphonema zellense* (0.764, *p* = 0.008), *Nitzschia filiformis* (0.714, *p* = 0.030), *Pinnularia viridiformis* (0.764, *p* = 0.004) and *Stauroneis anceps* (0.730, *p* = 0.041).

The comparison of functional trait frequencies between CP and GS revealed significant differences for several key traits (Table S9, Fig. 6b). Low-profile forms (LowPro) were significantly more frequent in GS (mean frequency: 5.17 vs. 1.17 in CP; *p* = 0.003), and taxa with mucilaginous tubes (MucoTub) were also more abundant in GS (2.00 vs. 0.08; *p* = 0.003). Mobile taxa exhibited higher frequencies in GS (19.50 vs. 8.42; *p* = 0.004), as did benthic taxa (19.50 vs. 8.42; *p* = 0.006). Among attachment traits, high attachment (ATT_Hig) was more common in GS (3.50 vs. 1.00; *p* = 0.005), as was ATT_Low (14.25 vs. 11.00; *p* = 0.005). Biovolume classes showed notable differences: BioVol_c2 was enriched in GS (10.00 vs. 4.00; *p* = 0.005), while BioVol_c5 was slightly more frequent in CP (2.42 vs. 2.33; *p* = 0.04). Motile forms tended to be higher in CP (13.17 vs. 9.50; *p* = 0.08), although this difference was marginally significant. Other traits, including medium attachment (ATT_Med), high-profile forms (HighPro), pedunculate forms, small biovolumes (BioVol_c1, c3), planktonic, colonial, and pioneer taxa, did not differ significantly between sites. Overall, GS communities are characterized by higher frequencies of small, mobile, low-profile, and mucilaginous-tube taxa, whereas CP shows relatively higher representation of certain larger or motile forms, reflecting distinct functional trait compositions between coastal and mountain environments.

### Species-Trait Network

**Fig. 7:**
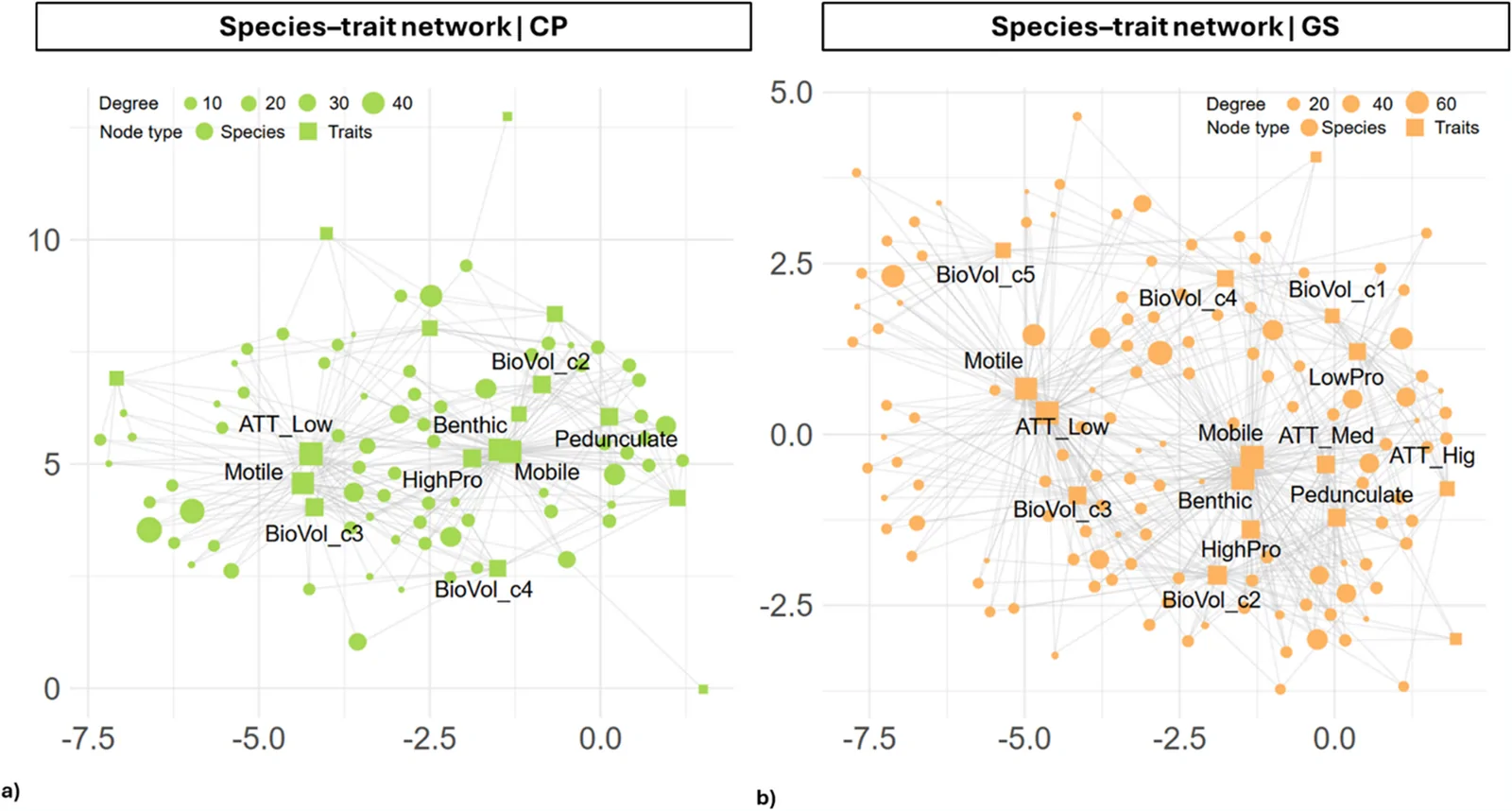
Bipartite networks showing the relationships between species and functional traits for the two study sites. Nodes represent species (circles) and traits (squares), with node size proportional to the degree (number of connections). Edges indicate the presence of a trait in a given species. Only the most connected nodes are labeled for clarity. Networks are shown separately for (**a**) Castelporziano (green nodes) and (**b**) Campo Imperatore (orange nodes)

The bipartite species–trait networks revealed differences in the structural properties of diatom assemblages between CP and GS (Fig. 7a and b). In CP, the network exhibited a modularity of 0.32 and a low density of 0.079, indicating a moderately modular but sparse network. The most highly connected nodes included traits related to low attachment (ATT_Low, degree = 48), benthic lifeform (Benthic, 42), mobility (Mobile, 42), motility (Motile, 41), and high-profile guild (HighPro, 20), suggesting that these traits are shared among many species. In GS, the network was slightly less modular (modularity = 0.29) and sparser (density = 0.056). Highly connected nodes included Mobile (77), Benthic (75), ATT_Low (75), Motile (65), and BioVol_c2 (38). Permutation tests based on 999 randomizations of the species–trait networks confirmed that the observed modularity in both CP and GS was significantly higher than expected by chance (CP: *p* = 0.001; GS: *p* = 0.003), demonstrating non-random clustering of species–trait associations in both regions. Overall, these results indicate that functional traits are structured in a non-random, modular manner, with certain traits acting as central connectors within the species–trait network, highlighting the presence of key functional hubs that potentially shape community assembly.

## Discussions

The analysis presented here provides an important starting point for understanding and interpreting ecological differences in diatom communities inhabiting temporary ponds (TP). As shown in the literature for other organisms [68], elevation and the biogeographical settings of sites can strongly shape the composition of freshwater communities [69]. Although research on this topic remains limited, our findings highlight that environmental variation at the biogeographical scale could influence both the structure and functional organization of diatom communities. This underscores the ecological relevance of these fragile habitats which sustain unexpectedly diverse communities.

To better interpret our findings, it is important to consider the seasonal timing of sampling (February in CP vs. July in GS). During winter, productivity in Mediterranean ponds may be limited by lower temperatures, reduced solar radiation, and decreased hydrological stability, which could constrain diatom growth [31]. In contrast, Alpine ponds in summer are characterized by higher light availability and enhanced photosynthetic activity [10], stable conditions that coincide with seasonal peaks in diatom diversity [45], also favored by mineral inputs from snowmelt. Finally, the alpine sites are embedded in a high elevation context where species pools may be broader and ecologically distinct compared to Mediterranean regions [70]. Steeper environmental gradients in temperature, solar radiation, and photoperiod create a greater heterogeneity of available niches, potentially favoring the coexistence of multiple species [71].

### Taxonomic and Functional γ Diversity

The results obtained indicated clear differences in diatom γ diversity between the two sites, a pattern also observed in Mediterranean and Alpine regions for other biological groups [72]. Although TP from both study areas are defined under the same broad category of inland waters (*sensu* Habitat Directive, 92/43/EEC), they exhibited substantial differences in overall species richness. The current classification of TP, largely based on hydroperiod, overlook additional drivers such as climate, soil type, and geographic setting (both latitudinal and elevational), all of which can strongly influence freshwater bodies and their biotic communities [31, 73, 74]. In our case, samples from Castelporziano (CP) generally showed lower species richness compared to Campo Imperatore (GS). This result is unexpected: Mediterranean ponds, generally characterized by higher nutrient inputs [5], would be expected to support richer communities [75, 76]; Conversely, Alpine ponds are often subject to strong daily fluctuations in climatic, edaphic, and physicochemical conditions [10], together with reduced nutrient input, which typically make them less suitable for sustaining high biodiversity [77]. Furthermore, The Castelporziano estate, despite being fenced and inaccessible, receives substantial nutrient inputs, including anthropogenic ones, mainly from agricultural activities within the surrounding land [43]. This enrichment of soils and groundwater with nitrogen and phosphorus could elevate the trophic status of ponds, promoting higher diatom productivity and diversity [78]. By contrast, the mountain ponds analyzed are located in a markedly different context: although modestly affected by summer tourism [79], they remain distant from agricultural activities and direct anthropogenic pollution.

Furthermore, our results indicated that the diatom community in GS was not only more diverse on average per pond, but also exhibited faster species accumulation with increasing sampling effort compared to CP. This suggests that alpine ponds host less redundant communities, with higher variability between individual ponds, whereas Mediterranean ponds tend to host more similar assemblages. In February (CP), many species may be absent or occur at low abundance due to sub-optimal conditions, including limited light, lower temperatures, and unstable hydrology [80]. In July (GS), environmental conditions are more favorable and variable, allowing a wider range of species with differing ecological requirements to coexist, leading to greater spatial diversification [81]. The significantly higher AUC observed in GS indicates that the alpine metacommunity draws from a richer regional species pool. This pattern aligns with the biogeographic hypothesis, as mountain areas often support both specialized Alpine/Boreo-Alpine taxa and more generalist species, thereby increasing overall diversity [82–84].

These results were not confirmed by functional traits analysis, which revealed greater functional trait richness in the Mediterranean sites compared to the Alpine ones. Such decoupling between species richness and functional richness has been documented across taxa and ecosystems, generally occurring when additional species primarily occupy already-filled regions of trait space rather than expanding their boundaries [85]. Consequently, the higher species richness observed in GS does not necessarily translate into a broader functional spectrum.

In contrast, CP showed a less species-rich but more functionally diverse pool, potentially due to influences from multiple environmental contexts (coastal, brackish, and freshwater with strong gradients) [86]. High connectivity and dispersal, facilitated by the numerous animal species using TP as watering and breeding sites (birds, mammals, amphibians), could influence which species colonize the ponds and, consequently, the occupied functional space [43, 87]. If CP offers environmental conditions that support a wider range of strategies, such as more heterogeneous microhabitats, substrates, or chemical gradients, filtering may be less restrictive, allowing occupation of an expanded trait space. Meanwhile, GS may experience stronger environmental filtering, yet with many species that are functionally similar coexisting in overlapping niches. It is important to acknowledge that the observed differences in functional trait space between CP and GS may partly reflect our sampling strategy. Stones were collected exclusively along the shoreline, up to approximately 1 m from the water’s edge, where diatoms are most abundant. Consequently, species associated with open-water habitats or more central zones of the ponds, which might include more planktonic or motile forms, could be underrepresented. This spatial restriction may lead to an overemphasis on traits common to shoreline communities, such as attachment structures or low-profile morphologies, and could influence measures of functional richness, dispersion, and overlap. Therefore, while the patterns observed provide valuable insights into community structure, they should be interpreted in the context of this sampling limitation, as inclusion of the inner zones could potentially increase the functional diversity captured, particularly in larger or more heterogeneous ponds.

However, seasonal composition of diatom taxa and the related expression of functional traits can vary substantially. Studies have shown that Functional Richness (FRic) and other facets of functional diversity fluctuate seasonally in response to temperature, nutrient availability, and hydrology [88, 89]. Therefore, some of the observed differences may reflect temporal effects: CP, sampled in February, could host species with more extreme traits than GS in July [90]. Although Functional Dispersion (FDis) and Functional Divergence (FDiv) are similar between sites, the most plausible explanation is that CP includes rare or occasional species occupying peripheral regions of the trait space (increasing total FRic), while the overall distribution and relative abundance of traits remain comparable across both regions.

### Compositional and Functional Analysis

Our results showed that diatom communities differed significantly between sites (CP vs. GS), while internal variability within sites (i.e., dispersal among pools of the same site) was comparable. These findings indicate that observed differences were not due to greater heterogeneity within a site but rather reflect a distinct and consistent species compositions between the two environments. Furthermore, almost all of the observed β diversity arises from species turnover rather than nestedness. In other words, CP and GS hosted a distinct, site-specific community likely shaped by different environmental filters. This pattern underscores how temporary habitats in different biogeographic regions support unique diatom assemblages [91] Diatom diversity may be more influenced by local environmental factors than by broad-scale climatic variables such as elevation [39]. These differences may also reflect geographic separation rather than small-scale environmental variation [36, 92]. The absence of detailed physicochemical analyses in our study limits further interpretation in this context and highlights the need for more in-depth investigations.

Importantly, many studies on temporary or extreme habitats report that turnover is the dominant component of β diversity. Spatial patterns in freshwater microalgal communities are largely driven by turnover rather than nestedness, reflecting local environmental gradients rather than progressive extinction–colonization dynamics [93]. Furthermore, in diatom communities of temporary habitats diversity is primarily structured by environmental differences and ecological filtering rather than nestedness [94]. By contrast, in more stable systems such as permanent lakes or large rivers, nestedness often plays a larger role: stress gradients (e.g., nutrient availability, salinity) produce communities that represent predictable subsets of the regional species pool [95, 96]. The predominance of turnover in our study suggests local adaptation to divergent environmental conditions. In CP, communities are likely shaped by unstable hydrology, coastal organic inputs, and low seasonal temperatures [97]. In GS, communities are influenced by high-altitude conditions, intense radiation, and mineral inputs from snowmelt [98]. The comparable internal dispersal indicates that both systems exhibit similar local-scale variability: each pool differs from the others to a similar degree, yet the species pools themselves differ substantially between sites. The additional Raup–Crick β-diversity analysis indicates that community assembly in our system is shaped by a combination of stochastic and deterministic processes. The mean βRC value (0.56 ± 0.35) suggests that stochastic mechanisms, such as ecological drift and random dispersal, likely play the predominant role in driving community turnover. However, the relatively high variability among pairwise comparisons shows that deterministic forces, including environmental filtering or niche differentiation, also contribute, particularly to a subset of pond pairs that deviate toward more extreme βRC values.

This pattern is consistent with a scenario in which species turnover dominates but arises from a balance between neutral processes and environmental constraints, rather than being driven primarily by deterministic filters. Internal βRC values indicate comparable levels of within-site variability in both systems, meaning that local-scale processes structure communities similarly at CP and GS. At the metacommunity level, the distinct species pools of Mediterranean and Alpine environments continue to contribute independently to regional diversity [99]. Therefore, conservation strategies should consider both systems, as each protects complementary components of taxonomic and functional diversity.

From a functional point of view, however, the investigated diatom communities showed some functional redundancy: at CP, there was a tendency towards functional clustering, while at GS, there was both clustering and a slight overdispersion. At the regional scale, therefore, no significant differences emerged between sites, suggesting that functional patterns were mainly driven by specific local conditions, rather than by systematic environmental filters at the regional level. The overlap index further highlighted that most of the functional trait space was shared between regions, indicating that both sites host species within a largely common “global functional space,” with only minor differences attributable to traits unique to each location. Functional clustering (SES < 0) is often interpreted as a sign of environmental filtering [100]. In diatoms and phytoplankton, several studies have observed clustering in environments subject to strong physicochemical constraints, such as salinity, conductivity, water stress [58, 101], At local scale, diatom communities alternate clustering and overdispersion signals depending on environmental gradients, while at the regional scale they tend to converge on a common trait-space [102, 103].

In other freshwater systems, high functional overlap between regions has been interpreted as evidence of functional redundancy [104, 105], suggesting that similar ecological strategies are replicated across different environmental contexts. Functional redundancy in this case must be considered in light of the fact that, despite differences in environmental conditions, both systems consist of TP, where shallow water and highly variable ecological conditions select for similar functional traits. In other words, Alpine and Mediterranean TP draw from the same “catalogue of strategies,” though implemented by different taxonomic compositions. Finally, the presence of clustering signals in specific sites indicates that some functions may depend on a few key species selected by the environmental filters è [106, 107]. This implies that these habitats could be particularly vulnerable to environmental changes, making them susceptible to the loss of specific traits and functional homogenization [108]. Consequently, monitoring and conservation efforts should aim to preserve key microhabitats within each site, safeguarding both species and the unique functional traits they support.

### Indicator Species, Traits Analysis and Species-Trait Network

The results from the species- and trait-specific analyses align well with the characteristics of the studied sites. In GS, most species are typical of well-oxygenated, moderately productive, and stable benthic habitats, consistent with alpine conditions and with those reported in similar studies [45]. For instance, *Achnanthidium minutissimum* and *Planothidium frequentissimum* can be considered benthic generalists, tolerant but characteristic of fresh, oligotrophic waters [38, 109]; *Encyonema spp*. (*caespitosum*,* mesianum*,* minutum*) are epiphytic or mucilaginous species adapted to colonizing submerged surfaces and mobile substrates [110]; *Staurosirella pinnata* is a colonial diatom, often associated with oligotrophic conditions and fluctuating nutrient availability [111]; *Navicula spp*. (*cryptocephala*,* cryptotenella*,* trivialis*,* veneta*) are benthic species preferring fine substrates and perform well under moderate water stress [112, 113]; *Fallacia subhamulata* and *Cocconeis placentula* are typical of stable, well-lit substrates. Overall, these species describe GS as an environment dominated by small, specialized forms, highlighting the high ecological relevance and reliability of diatoms as bioindicators even in temporary Alpine habitats.

In CP, the analysis revealed greater heterogeneity, reflecting the more variable abiotic conditions of Mediterranean TP. Here, species were often associated with extreme or fluctuating environmental conditions and stress: *Eunotia bilunaris* and *E. minor* are typical of acidic or oligotrophic waters, indicating particular chemical stresses [114]; *Gomphonema zellense* is generally associated with substrates subject to environmental fluctuations and shows tolerance to variable conditions [115]; *Nitzschia filiformis*, a mobile species, is often linked to eutrophic or disturbed waters [116], while *Pinnularia viridiformis* and *Stauroneis anceps* are larger species, preferring oligotrophic environments and waters with distinctive geochemical characteristics [109, 117]. Overall, the species composition of CP suggests a habitat more exposed to chemical and hydrological stress, colonized by tolerant taxa, with a predominance of large benthic or mobile forms.

The analysis of functional traits reflected a certain redundancy in functional traits, although with some important differences. For example, the higher frequency of low-profile forms (LowPro) represents an adaptive strategy to resist mechanical disturbance (e.g. turbulence, frost, partial desiccation) [58]; Taxa with mucilaginous tubes (MucoTub) favor anchoring in variable conditions and allow the colonization of stable microhabitats [118]; a higher frequency of small biovolumes (c2) is instead an adaptation to oligotrophic conditions, high efficiency in nutrient absorption [119, 120]. Overall, these traits describe resilient and plastic communities, capable of surviving in cold and oligotrophic environments, exploiting anchoring and miniaturization strategies. Conversely, species in CP showed a higher frequency (albeit marginal) of motile forms, which can represent an advantage in more variable and disturbed environments, where mobility allows for the exploration of different microhabitats and is typical of temporary habitats [25, 121, 122]. CP also showed a relatively higher occurrence of large biovolume species (c5), which, while less efficient under oligotrophic conditions, can confer competitive advantages in unstable or episodically nutrient-rich environments [116]. Overall, the CP community reflects a more opportunistic strategy, characterized by mobility and larger body size, enabling rapid responses to environmental fluctuations.

Network analysis confirmed that species–trait associations in our sites reflect a well-defined functional organization. In CP, species tend to cluster into distinct functional modules, but the connections between species and traits were relatively few, indicating a more “compartmentalized” structure. Such structuring suggests that environmental conditions select for specific niches, resulting in clearer and more separate functional subgroups. In contrast, the network in GS was less modular and sparse, producing a more fragmented community in which traits and species are distributed less homogeneously. This pattern indicates that species share traits more redundantly, potentially as a resilience strategy under extreme climatic conditions [123, 124]. Node analysis further revealed that, in CP, the functional structure revolves around mobile forms with weak attachment (low attachment) and a notable proportion of high-profile species, consistent with an opportunistic strategy that allows rapid responses to environmental variability [58, 125]. In Alpine sites, by contrast, traits are concentrated in small, mobile species, reflecting low nutrient availability and the need for active movement to exploit favorable microhabitats. Diatom communities of extreme environments are dominated by small, mobile forms adapted to nutrient-poor and physically extreme conditions [126]. Although the application of network analysis to diatom communities is relatively novel, studies in other systems support our findings: functional traits in communities are not randomly distributed but tend to form clusters or modules shaped by environmental conditions and ecological filters [105]. Furthermore, in ecological networks generally reflect adaptive functional strategies, highlighting the ecological significance of non-random trait organization [127].

## Limitations of the Study

Although this study was conducted rigorously following established protocols for both field sampling and data analysis, several limitations should be acknowledged for scientific transparency. First, a relatively small number of TP were sampled in the Campo Imperatore region. However, all accessible and water-containing ponds were included, based on repeated field surveys conducted from May to October, which did not reveal additional suitable sites. Second, due to geographical and ecological constraints, sampling was carried out in different seasons for the two regions: winter for Mediterranean ponds and summer for Alpine ponds. Seasonal differences in water availability influenced accessibility; in summer, many ponds in Castelporziano were dry, whereas in winter, ponds in Campo Imperatore were often frozen or covered by snow, making field access logistically challenging. Finally, environmental and abiotic parameters such as physical and chemical variables, chlorophyll concentrations, nutrient levels, hydroperiod, and soil properties were not measured. These factors can substantially affect diatom biodiversity and functional organization. Due to financial constraints and limited resources, these analyses could not be included in the present study.

## Conclusions

Temporary ponds, fragile and understudied ecosystems, actually host remarkable biodiversity and play a crucial role in maintaining ecological balance. In this study, we showed that TP in different biogeographical regions can host distinct communities, focusing specifically on diatoms. Our results revealed that γ diversity differed markedly between the two study sites, with alpine ponds exhibiting higher taxonomic and functional richness. These differences between biogeographic regions were also reflected in the composition of diatom communities, indicating that each site supports unique assemblages. Despite these regional differences, within-site community dispersion did not differ significantly. Community-level analyses further indicated that, although functional trait distributions were largely similar across sites, local environmental constraints can generate site-specific clustering, underscoring the importance of fine-scale environmental heterogeneity in shaping functional community structure. Notably, a certain degree of trait space was shared between the two regions. Overall, diatom communities in the mountain sites were characterized by higher frequencies of small, mobile, low-profile, and mucilaginous-tube taxa, whereas coastal ponds displayed relatively higher representation of larger or motile forms, highlighting distinct functional compositions between mountain and coastal environments.

While our study represents an initial exploration and did not cover all environmental features, it provided an important base for future research on diatom communities in temporary habitats. In the context of ongoing and rapid climate change, understanding these patterns is essential, as community structures may shift or decline, potentially leading to irreversible losses in both taxonomic and functional diversity.

## Supplementary Information

Below is the link to the electronic supplementary material.

Supplementary File 1 (XLSX 176 KB)

## Data Availability

The datasets used and/or analyzed during the current study are available from the corresponding author on reasonable request.
